# Emerging Advances of Nanotechnology in Drug and Vaccine Delivery against Viral Associated Respiratory Infectious Diseases (VARID)

**DOI:** 10.3390/ijms22136937

**Published:** 2021-06-28

**Authors:** Amir Seyfoori, Mahdieh Shokrollahi Barough, Pooneh Mokarram, Mazaher Ahmadi, Parvaneh Mehrbod, Alireza Sheidary, Tayyebeh Madrakian, Mohammad Kiumarsi, Tavia Walsh, Kielan D. McAlinden, Chandra C. Ghosh, Pawan Sharma, Amir A. Zeki, Saeid Ghavami, Mohsen Akbari

**Affiliations:** 1 Laboratory for Innovations in Micro Engineering (LiME), Department of Mechanical Engineering, University of Victoria, Victoria, BC V8P 5C2, Canada; amirseyfoori@uvic.ca (A.S.); tavia.r.walsh@gmail.com (T.W.); 2 Biomaterials and Tissue Engineering Department, Breast Cancer Research Center, Motamed Cancer Institute, ACECR, Tehran 1517964311, Iran; 3 Department of Immunology, Iran University of Medical Sciences, Tehran 1449614535, Iran; mahdie_shokrollahy@yahoo.com; 4 ATMP Department, Breast Cancer Research Center, Motamed Cancer Institute, ACECR, Tehran 1517964311, Iran; 5 Department of Clinical Biochemistry, School of Medicine, Shiraz University of Medical Sciences, Shiraz 7134845794, Iran; mokaram2@gmail.com; 6 Autophagy Research Center, Health Policy Research Center, Institute of Health, Shiraz University of Medical Sciences, Shiraz 7134845794, Iran; 7 Department of Analytical Chemistry, Faculty of Chemistry, Bu-Ali Sina University, Hamedan 6517838695, Iran; m.ahmadi@basu.ac.ir (M.A.); madrakian@basu.ac.ir (T.M.); 8 Influenza and Respiratory Viruses Department, Pasteur Institute of IRAN, Tehran 1316943551, Iran; mehrbode@pasteur.ac.ir; 9 Department of Pharmaceutical Nanotechnology, Faculty of Pharmacy, Tehran University of Medical Sciences, Tehran 14155-6451, Iran; klm.1985@yahoo.com; 10 Department of Human Anatomy and Cell Science, Rady College of Medicine, Max Rady Faculty of Health Sciences, University of Manitoba, Winnipeg, MB R3E 0J9, Canada; m.kiumarsi@gmail.com; 11 Department of Laboratory Medicine, School of Health Sciences, University of Tasmania, Launceston, TAS 7248, Australia; kielan.mcalinden@utas.edu.au; 12 Roger Williams Medical Center, Immuno-Oncology Institute (Ix2), Providence, RI 02908, USA; chandrachurghosh@gmail.com; 13 Center for Translational Medicine, Division of Pulmonary, Allergy and Critical Care Medicine, Jane & Leonard Korman Respiratory Institute, Sidney Kimmel Medical College, Thomas Jefferson University, Philadelphia, PA 19107, USA; pawan.sharma@jefferson.edu; 14 Division of Pulmonary, Critical Care, and Sleep Medicine, Department of Internal Medicine, U.C. Davis Lung Center, Davis School of Medicine, University of California, Davis, CA 95817, USA; aazeki@ucdavis.edu; 15 Veterans Affairs Medical Center, Mather, CA 95817, USA; 16 Biology of Breathing Theme, Children Hospital Research Institute of Manitoba, University of Manitoba, Winnipeg, MB R3E 0J9, Canada; 17 Research Institute of Oncology and Hematology, Cancer Care Manitoba, University of Manitoba, Winnipeg, MB R3E 0J9, Canada; 18 Biotechnology Center, Silesian University of Technology, Akademicka 2A, 44-100 Gliwice, Poland; 19 Center for Advanced Materials and Related Technologies, University of Victoria, Victoria, BC V8P 5C2, Canada

**Keywords:** viral infection, SARS-CoV-2, nanomedicine, respiratory disease, nano-vaccine, COVID-19

## Abstract

Viral-associated respiratory infectious diseases are one of the most prominent subsets of respiratory failures, known as viral respiratory infections (VRI). VRIs are proceeded by an infection caused by viruses infecting the respiratory system. For the past 100 years, viral associated respiratory epidemics have been the most common cause of infectious disease worldwide. Due to several drawbacks of the current anti-viral treatments, such as drug resistance generation and non-targeting of viral proteins, the development of novel nanotherapeutic or nano-vaccine strategies can be considered essential. Due to their specific physical and biological properties, nanoparticles hold promising opportunities for both anti-viral treatments and vaccines against viral infections. Besides the specific physiological properties of the respiratory system, there is a significant demand for utilizing nano-designs in the production of vaccines or antiviral agents for airway-localized administration. SARS-CoV-2, as an immediate example of respiratory viruses, is an enveloped, positive-sense, single-stranded RNA virus belonging to the coronaviridae family. COVID-19 can lead to acute respiratory distress syndrome, similarly to other members of the coronaviridae. Hence, reviewing the current and past emerging nanotechnology-based medications on similar respiratory viral diseases can identify pathways towards generating novel SARS-CoV-2 nanotherapeutics and/or nano-vaccines.

## 1. Introduction

Viral infectious diseases and respiratory viral infections are among the most severe global health threats. According to the World Health Organization (WHO), millions of people are globally affected by viral diseases annually [1]. VRIs caused by different viral sources can infect the human upper and lower respiratory tracts, making the respiratory mucosa the primary gate of entry. Many viruses that could potentially lead to VARID have been reported in the current literature. Table 1 summarizes these viruses and their associated respiratory infectious disease. In this regard, SARS-CoV-2 and the influenza virus H1N1 are among the most recent to have caused global pandemics. 

Although pathogens of viruses in the respiratory system are different, the most common associated and fatal viral infections have specific tropism to the lower respiratory tract, causing severe pneumonia and acute respiratory distress syndrome (ARDS) [2]. The susceptible epithelial cells express the specific surface proteins by which they bind the viruses. The expression pattern and distribution of these receptors in the epithelial layer of the respiratory system can be used to determine the virus localization [3]. As a current example, SARS-CoV-2 binds to angiotensin-converting enzyme type 2 (ACE-2), which is overexpressed in intermediated and lower regions of the respiratory tract [4]. The infection lesions of SARS-CoV-2 are not common in the mouth and sinuses [5]. Normally, the immune system can trap the viral particles in the mouth, nose, and upper respiratory tract at the onset of the virus entry, inhibiting penetration into the lower parts of the lungs. However, the most important concerns about SARS-CoV-2 as a viral respiratory infection are pulmonary insufficiency, as categorized in viral associated respiratory infection diseases (VARID) [6]. While the infection susceptibility and disease progression are directly related to the ability of the immune system to combat the viruses, the dysregulation of immune system responses also has an impact on increased inflammation, destruction of alveoli, and obstruction of the airways (immunopathogenesis). Immunopathogenesis can justify the occurrence of viral infection lesions due to over-activation of the immune system [7]. Current anti-viral pharmaceutics suffer from several shortcomings, including the development of drug resistance, non-targeting of the particular virus inside the host cell without causing adverse cellular effects, and the lack of a generic anti-viral drug suitable for a wide range of viruses [8,9,10]. Respiratory anti-viral treatment using nanocarriers can be conducted through either systemic interventions [11], or localized methods [12]. The deep penetration, targeted delivery, and higher stability are the most prominent features of efficient treatments in SARS-CoV-2 (as an incurable VARID); nanotechnology-based interventions may provide these features. Hereby, the importance and necessity of using nano-pharmaceutical therapies and nano-vaccines against severe respiratory infections are caused by a range of viruses become evident. Besides the impact of nano-carriers on anti-viral therapeutics, they also play an essential role in the development of new generations of the nano-vaccines [13]. Nano-vaccines may facilitate a more effective vaccination process through enhanced stability, protection from premature degradation, and by assisting adjuvant and/or targeted delivery agent of an immunogen to the particular antigen-presenting cells (APCs). The benefits of nanoparticle administration in the lower respiratory tract include the following: Uniform drug distribution and enhanced drug solubility; sustained release of anti-viral drugs or immunoactive API into targeted mucosal system cells, and; host cell or immune cell uptake through phagocytosis. These features have made nanocarriers into specific nanotherapeutic candidates in respiratory diseases and, in particular, VARID [14].

In this review, the innate immune system responses to different VRIs and nanotechnological approaches to combating the coronavirus family are discussed. We, then, conduct a holistic review of the past evolutions and current opportunities of nanotechnology in respiratory viral disease treatments and active immunizations.

## 2. Anti-Viral Responses of the Immune System in VARID and Evidence of Nanomedicine

In this section, the immune system framework is considered in the case of interventional nano-medical tools. According to the latest findings in nanomedicine, the treatment strategies would be focused on synergizing anti-viral targeted therapy or enhancing the immunomodulation responses against exaggerated anti-viral responses that lead to viral infection associated immunopathogenesis. There are three general immunological approaches in viral respiratory infections, including physical-mucosal barrier (innate immunity), natural, and secondary IgA and cell mediated immunity (adoptive immunity). The barriers from the perspective of the nanomedicine interventions are discussed.

### 2.1. Physical-Mucosal Barriers from Saliva to Bronchus-Associated Lymphoid Tissue

The physical-mucosal barriers from the oral and nasal cavities to the deepest regions of the lungs are considered the first line of defense in the innate immune system [22]. Alongside these physicochemical barriers, scattered lymphatic regions in the basal side of the respiratory tract (e.g., Nasal Associated Lymphoid Tissue (NALT) in the nasal cavities and mucosal associated lymphoid tissue in the mucosal layer of the respiratory tract) have a critical role in tropic anti-viral immunity responses [23]. Stimulation or suppression of local immune responses in the respiratory tract are the basis of many vaccines or immunomodulatory drugs. For example, intranasal vaccines can boost IgA production and block the virus entry. Nanoparticles in such drugs and/or vaccines play the role of scaffold, retainer, emulsifier, stabilizer, targeted, and scheduled drug release carrier [24]. In this regard, nano-formulated combinations, such as Flu Avert^®^ and FluMist^®^ are designed as effective intranasal influenzas vaccines [25]. Moreover, intranasal interventions are also used for local drug delivery, particularly in drug delivery to the lungs, administered using intranasal droplets or sprays.

The oral cavity is another physical barrier with enzyme-containing saliva and other NALT layers [26]. The contents of the saliva and nose mucosa in the respiratory tract determine the type of immune response. Given that the mouth and nose are the primary gateway for virus entry into the pulmonary tract, passing the virus through the saliva barrier, nasal mucosa, and ciliated layers of the upper respiratory tract can lead to acute viral infection in the lungs [27]. Some periodontal therapies using silver and gold nanoparticles can improve the immunity of the oral cavity and prevent the penetration of pathogens into the epithelium [28].

Saliva content, as an element of oral immunity, includes peptides, enzymes, and immune/epithelial cells derived cytokines. Host defense peptides (HDP or antimicrobial peptides (AMP)) of the innate immune system, such as Cathelicidin (LL-37), α, β defensins, lactoferrin, lysozyme, and heterotypic salivary agglutinin (gp340, DMBT1) are secreted from the epithelial cells of the mouth, can consequently block the virus entry into epithelial cells or inhibit virus pathogenesis [29]. Rhesus theta-defensin 1 (RTD-1) is a new cyclic defensin that has a prophylactic effect on SARS-CoV infection and prevents death in animal models [30]. It is decorated in dendrimer-based nanoparticles and acts to enhance the antiviral function [31]. APCs, specifically Langerhans cells, can migrate to the epithelium due to local infection, uptake antigens, and return to the lymphoid tissues to activate T cells and induce B cell differentiation into plasma cells [32]. The Langerhans cell activation in the oral cavity and small intestine is the essential basis of IgA, inducing vaccine design. According to this, some of the nano-formulated vaccines can boost the mucosal immune responses and raise the specific IgA titration, depending on the adjuvant decoration and administration routes. For example, colloidal saponin containing micelle liposomes, or immune-stimulating complexes (ISCOMs), with a size of approximately 40 nm are considered the most prevalent self-adjuvants in oral nano-vaccine formulations [33] (26).

One of the most important AMPs in saliva and mucus is Cathelicidin (LL37); known for its antimicrobial role via lipopolysaccharide (LPS)-binding function, the anti-viral impacts were recently established in rhinoviruses and influenza [34,35,36]. Although saliva-derived HDPs and other antimicrobial elements might be used in the formulation of some nanoparticles, the saliva content can impact the fate of oral nanoparticles [37]. Nowadays, some nano-antibiotics have been developed, containing membrane-active human LL37 and synthetic compounds that mimic antimicrobial peptides, such as ceragenins [38]. CSA-13 is a ceragenin with an anti-viral effect on viral DNA replication in vaccinia virus and smallpox virus [39]. While magnetic nanoparticles containing LL37 (MNP@LL37) and CSA-13 (MNP@CSA-13) can increase the antimicrobial potency of LL37 and cathelicidin [40], carbon nanoparticle decoration of these compounds reduces this effect through structure alteration and affinity variation [41,42]. Synthetic LL37 has been found to inhibit spike protein binding to ACE2 in vitro in SARS-CoV-2 infections [43]. Since LL37 is induced after Vitamin D uptake, cathelcidin was measured after Vitamin D intervention in SARS-CoV-2 patients (NCT04636086) [44]. Conjugation of LL37 with carbon nano tubes (CNTs) facilitated the binding of LL37 on monocytes and accelerated their stimulation [45]. A large number of the most important mediators in innate immunity act as activating ligands of scavenger receptors expressed on APCs. The scavenger receptors are classified into three categories (SR-A, SR-B, and SR-C) and contain a highly glycosylated extracellular domain. This extracellular domain can interact with pathogen lipoproteins and liver circulating HDLs and LDLs. Studies have shown that silver nanoparticles (Ag-NPs) mimicking HDL are effective in activating the scavenger receptor pathway [46]. It has also been found that polyvinylpyrrolidone (PVP) -formulated Ag-NPs, such as nanowires and silver-coated plates can enhance the uptake of nanoparticles by mast cells and subsequently impact inflammation in the respiratory tracts in allergy models [47]. Sundararaj et al. worked on PVP-AgNPs to demonstrate the inhibitory function of these nanoparticles to the entry of SARS-CoV-2 in VeroE6/TMPRSS2 cells. However, it is strongly suggested that antiviral sprays inhibit virus entry [47].

**Figure 1:**
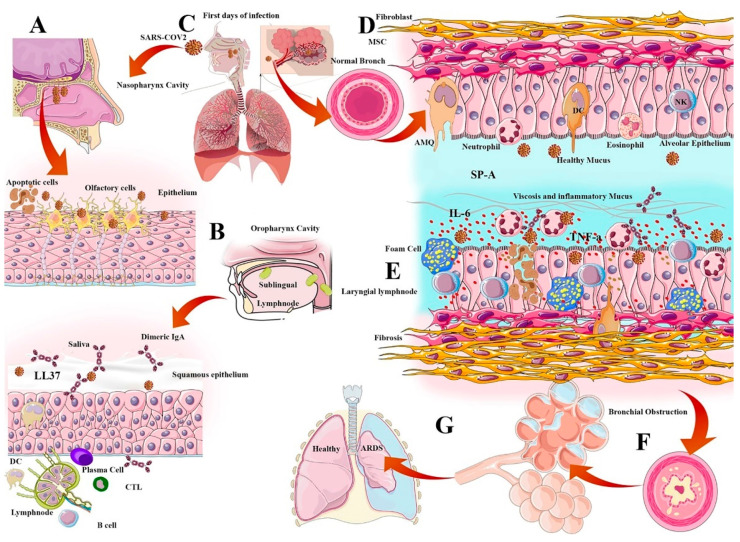
(**A**) Nasal virus entry homing in the nasopharynx cavity and virus attachment on epithelial cells and olfactory neurons. Virus replication in olfactory cells can decrease the ability of smell sensing and cause inflammation in the nasopharynx. (**B**) Oral cavity, salivary component including dimeric IgA, cathepsins, and sublingual and laryngeal lymph nodes are the first line of lymphoid tissue and antibody production. (**C**) Oral-nasal virus entry, oropharynx cavity, and virus attachment on the epithelial cells of throat. (**D**) Normal alveoli in first days of virus entry: thin layer of fibroblasts, low density and distribution of immune cells in a single epithelial layer, eosinophil and neutrophils number in a normal range. (**E**) Severe infection in the alveolar region: macrophages became foam cells. Inflammatory agents induce mucus secretion and increase the viscosity of the mucosal barrier. Alveolar epithelial cells die via apoptosis or viral cytolysis, NK cells increment, and neutrophils induce a cytokine storm. (**F**) Inflammatory conditions induce fibrosis and fibroblast cells proliferation, which can cause thickness of the alveolar cavity, resulting in respiratory distress. (**G**) Lung obstruction results in decreased respiratory rate.

Alveolar macrophages (AMQ) are the most prevalent immune cells located in all parts of the respiratory tract. AMQs stabilize the hemostasis of the alveolar regions through rapid recognition of infections and activation of immune responses, such as DCs, T cells, and B cells. AMQ have an important immunomodulatory role in mucosal immunity and can cause the cytokine storm initiation via TNF-α, IFN-g, and IL-6 production, or cascade repair beginning through TGF-β and culminating in IDO release due to viral infection [48]. The pathogenesis of IL-6 and AMQs in alveolar thickness and fibrosis induction is important for immunopathogenesis in VARIDS, which is described in SARS-CoV-2 infection and illustrated in Figure 1. Recent studies have concentrated on AMQ targeting nanoparticles and support virus grabbing agents such as decoy nanoparticles. These nanoparticles are made of vesicles containing receptors such as ACE2, IL6R, and GMCSFR, and can cause beneficial dampening of inflammation via the virus capturing and inflammatory cytokines [49].

Inducible Bronchial Associated Lymphoid Tissue (iBALT) are a subgroup of MALT that are generated due to viral infections in the extremities of the lungs. iBALT initially helps to produce a specific immune response and increase the rate of chemotaxis of immune cells. Some nanoparticles, such as protein cage nanoparticles (PCN), can moderate the iBALT function or increase the protection against respiratory viruses via macrophages and T cell chemotaxis increment [50]. iBALT induction is a critical aspect of vaccination in respiratory diseases. However, in some cases, due to the progression of the disease, it can exacerbate the pathogenesis of the immune cells by increasing inflammation in the alveolar areas. Finally, more mucus production, increased inflammation, and exacerbation of fibrosis will cause narrowing of the alveoli, reduced respiratory saturation, and subsequent ARDS [51]. Reactive oxygen species (ROS) release incrementally by immune cells and AMQs, producing inflammatory cytokines, such as TNFα and IL-6, thus, increasing the inflammation level and causing poor prognosis as the immunopathogenesis of VARID leading to ARDS. Some immunomodulatory treatments based on nanoparticles, such as piceatannol incorporated albumin nanoparticles (PANPs), can be used to reduce ARDS through neutrophils adherence targeting [52]. For SARS-CoV-2, anti-inflammatory nanoparticles such as silver and gold nanoparticles are very important and can change the microenvironment in favor of immunomodulation [53]. Other metal oxide nanoparticles, especially ZnO combinations, reduce inflammation through ROS reduction [54].

### 2.2. Surfactant Role in Viral Infection

Pulmonary surfactant is a layer of phospho-lipoprotein compound secreted by Type 2 alveolar epithelial cells that covers the surface of the alveoli. This surfactant layer has amphipathic properties and traps water in the mucosal barrier to regulate the alveoli size and tension. There are four important functions of surfactants in anti-viral immunity and homeostasis, including anti-viral immunity enhancement, inhibition of viral infectivity, inflammation regulation, and virus entry facilitating. The pulmonary surfactants are categorized into four subtypes (SP-A, SP-B, SP-C, and SP-D) depending on their hydrophobic features and the protein contents. Generally, SP-B and SP-C are more hydrophobic and smaller, while the anti-viral immunity of surfactants is more related to SP-A and SP-D. These compounds can bind to the viral glycoproteins and facilitate phagocytosis; the loss of SP-A would lead to delayed virus clearance. The amphipathic properties of these surfactants make them suitable candidates for anti-viral nanoparticle decoration [55].

While surfactant-based vesicles containing dipalmitoyl phosphatidylglycerol (DPPG) can inhibit the viral infection, others have been shown to facilitate viral host cell entry [56]. Dipalmitoyl phosphatidylcholine (DPPC) is one of the surfactant components that can facilitate adenovirus entry into epithelium, thus would make a good candidate for lung disease gene therapies [57].

The anti-inflammatory features of these surfactants would also be exploited via binding to the signal inhibitory peptide (SIRP-α) on macrophages, causing them to attenuate the release of pro-inflammatory products. Currently, exogenous surfactants are administrated to diminish inflammation in ARDS patients. This exogenous surfactant may be synthetic like Surfaxin^®^, natural (bovine-derived) like Beractant (Survanta^®^), or nano-formulated. The injection route is also different in many trials: It is executed intrathecal, bronchoscopically, or aerosolized [58]. Clinical trials for the assessment of the efficacy of Lucinactant and other generic commercial brands in treating COVID-19 patients are ongoing. Anionic phospholipids like palmitoyl-oleoyl-phosphatidylglycerol (POPG) and phosphatidylinositol (PI) may inhibit TNF-α production and suppress the activity of macrophages in pro-inflammatory production [59]. The most common use of synthetic surfactants is in premature infants for the treatment of respiratory syndrome, as well as in severe cases of respiratory viral infections for lung recovery [60].

### 2.3. Antiviral IFN Route

It seems that, either an appropriate underlying genetic background showing a specific anti-viral response, or the utilization of anti-sera or PEGylated IFNα to stimulate the immune response, is significant at the incubation stage in people infected with SARS-CoV-2. The response to Type I interferon in the patients with poor prognosis has been significantly lower than that in the recovered patients during adaptive immune responses. When using a Type I IFN for treatment in a mouse model of SARS-CoV or MERS-CoV infection, the timing of administration is essential to obtain a protective response. During this type of response, CD4+ and CD8+ memory T cells can be stored for an extended period of time even if there is no antigen present and can induce the proliferation of T lymphocytes (the type of hypersensitivity response, delayed (DTH), and the production of IFN-γ, as found in the blood). Recent research showed that, while the CD8+ T cell response is crucial in individuals recovered from SARS-CoV-2, it must be well-monitored, in order to avoid lung inflammation [61,62]

**Figure 2:**
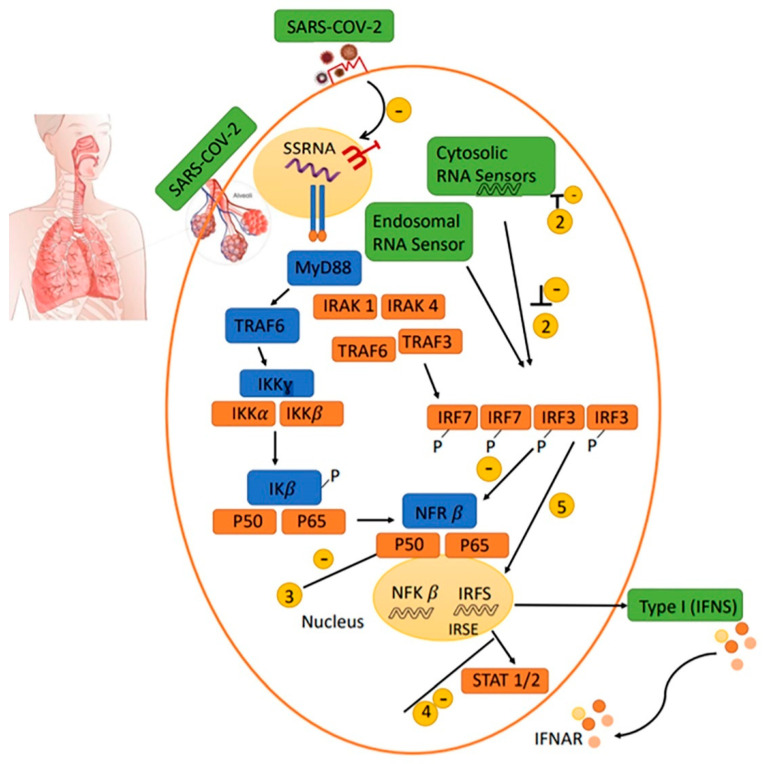
Proposed immune escape mechanism of SARS-CoV, MERS-CoV and possibly SARS-CoV-2. SARS-CoV-2 is attached to its receptor on the surface of target ACE2 positive cells, such as alveolar or other target cells, reducing the anti-viral IFN responses, leading to viral replication and propagation. COVID-19 may inhibit the pathways induced by TLRs3, 7, and 8, which are expressed in the endosomes. The suppression of these molecules leads to dampening of NF-kB, IRF signaling cascades, and STAT1/2 function in the nucleus, which decreases in the production of Type I IFNs responses. Delayed Type I IFNs responses may trigger immune exhaustion and the invasion of neutrophils and monocytes/macrophages into the infected cell, which may lead to cytokine storms and Th2 type responses resulting in poor outcomes.

As shown in Figure 2, coronaviruses possibly suppress several steps while the initial innate immune response is addressed. Cytosolic RNA sensors (RIG-I/MDA5), production of Type I IFN responses, and activation of IFN attached to its receptor, will be inhibited. Prolonged late Type I IFN responses cause immune collapse, which leads to poor prognosis of patients infected with SARS-CoV-2 [62].

### 2.4. Natural and Secondary IgA

Although IgG, IgM and IgA have an important role in the systemic humoral immunity, one of the most important compartments of mucosal immunity that can regulate lung hemostasis is the dimeric secretory immunoglobulin Type A that is released into the mucosal layer of the respiratory tract, saliva, and nasal mucus [63]. Secretory IgA, as another oral immunity content in the saliva, is produced by the lamina properia resident/induced plasma cells, which can pass the epithelium and concentrate in saliva. This primary secreted IgA can bind the virus-specific proteins, block the virus entry, and inhibit epithelium infection, especially in oral-respiratory infections, such as Cytomegalovirus (CMV), influenza, and SARS-CoV-2 [64].

Dimeric IgA production can modulate the inflammation in the alveolar area and protect the respiratory epithelium from high inflammatory response-related damage. It occurs due to viral particles blocking and modulating the respiratory dysfunction, especially in chlamydia dependent infections in neonates [65]. The uptake of IgA-loaded nanoparticles especially in chitin/chitosan nanoparticles within the nasal membranes following intranasal administration shows passive immunity in some respiratory diseases. Chitosan (CS) -dextran sulphate (DS) nanoparticles potentially increase the IgA-loaded combinations into nasal membranes and are widely used in intranasal formulations [66]. Some DNA vaccines have coupling capabilities with poly-lactide-co-glycolide (PLGA) and boost IgA production against respiratory syncytial virus (RSV) in acute respiratory disease caused by RSV in children [67]. The antibody-conjugated nanoparticles have a very important effect on the durability, stability increment, and decrease of the serum metabolism rate of immunoglobulins [68]. DiagNano™, a silica magnetic nanoparticle, is designed as an anti-human IgA, IgG, IgM conjugated nanoparticle formulated for rapid test diagnostic kits and can be used for other clinical approaches. PreveCeutical^®^, as a SiO_2_ sol-gel delivery platform, is an FDA approved nanoparticle used for nose-to-brain drug delivery [69] (63). The most effective vaccine routes in the COVID-19 pandemic may be inhaled vaccines, as this type of vaccine produces very powerful mucosal dimeric IgA that can block the virus entry in the first line of infection defense [70,71].

### 2.5. Cell Mediated Immunity (CMI)

Cell-mediated immunity (CMI) is a specific immune response for the destruction of cells infected with viruses and subsequently protects the body against cancers, fungi, protozoa, and bacteria. Generally, virus-infected cells activate CMI, causing CD4 or T helper cells to affect the appearance of phagocytes, antigen-specific cytotoxic T lymphocytes (CTLs), and the secretion of various cytokines against the antigen. CTL activation is dependent on DC interactions and antigen presentation. In this regard, some polymeric nanoparticles such as PEI-coated PLGA NPs can stimulate the specific DC generation to stimulate specific anti-viral CTL [72].

Besides T cell activation during viral infection, viruses can also stimulate the production of α-interferon from macrophages, which improves the function of natural killer cells and restrains the increase of viruses in neighboring cells. In this point, hyaluronic acid-gold nanoparticle/α-interferon complexes (HA–AuNP/IFNα) were used to increase the anti-viral response due to IFN-α stabilization and virus replication inhibition [73]. Additionally, NK cells can regulate the degree and duration of immune responses generated by T cells, B cells, macrophages, lymphocytes, and neutrophils. NK cells can also intervene antibody-dependent cell-mediated cytotoxicity (ADCC), enabling ADCC to intervene against cells infected with the virus. When IgG binds to antigens specific to a virus on the surface of an infected cell, the Fc portion becomes a target for effector cells paving the way for ADCC to intervene and bring about the lysis of the infected cell.

## 3. Anti-Viral Systemic or Local Nano-Vaccination and Immunotherapy

Vaccination is a general strategy for the control of infectious diseases and is considered a significant choice for fighting viral diseases [74]. Due to several limitations (e.g., failure to trigger the immune system, potential of high toxicity, invasive administration, low in vivo stability, storage, and transport temperatures requirement), the clinical outcomes of some vaccines against different viral infections are not significant enough [75]. However, with emerging new formulations of vaccines (i.e., nano-vaccines), many of the shortcomings of conventional vaccination protocols are successfully addressed. Nano-vaccines can induce and enhance both humoral and cell-mediated immune responses in a more effective way than their former generations [76].

As mentioned previously, NALT acts as a crucial defense barrier against respiratory viruses in the nasopharyngeal cavity [77] and provides a site for humoral and cellular immune responses, thus, representing a promising target for nano-vaccines against respiratory viruses [74]. Generally, nano-vaccination carriers include inorganic and polymeric nanoparticles, virus-like particles (VLPs), liposomes, and self-assembled protein nanoparticles [78]. These nano-vaccines can mimic specific mucosal immune responses by providing a distinct formulation, size, and antigen exposition similar to respiratory viruses [78,79,80]. VARID nano-vaccines lead to a specific immune response using inactivated pathogens, attenuated virus, or subunit protein antigens. Examples of inactivated virus vaccine formulations for seasonal respiratory diseases, such as influenza include Influvac^®^ [81], Vaxigrip^®^ [82], and Fluzone^®^ [83] against influenza Type A and Type B viruses. Examples of attenuated virus vaccine formulations include Nasovac^®^ and Flumist^®^ [84,85]. During the ongoing SARS-CoV-2 pandemic, a significant number of vaccines have been designed using nanotechnology such as Pfizer^®^, Moderna, NovaVax, Sinopharm, Sanofi–GSK, and others.

In relation to the surface charge, the positively charged polymeric, metallic, inorganic, phospholipidic and protein-based nanoparticles have shown higher immune response stimulations, in comparison to their negatively charged counterparts [86,87]. These particles can physically encapsulate antigen or covalently conjugate to an antigen and facilitate a suitable platform for vaccine delivery [88]. The most common strategies in viral respiratory vaccine solutions are to encapsulate antigens/epitopes within the nanoparticles to protect the structure of antigens from proteolytic degradation, as well as to deliver the antigen/epitopes to APCs and NALT. Another efficient reported strategy is the conjugation of antigens or epitopes on the surface of the polymer nanoparticles through which the viral behavior is mimicked [88,89,90]. These antigens are classified based on the pathogen, such as VLP, mRNA of antigenic protein, full peptide of immunogenic antigen, and dsDNA of antigen. For all types of antigens, first APCs internalize the vaccine and trigger lysosomal destabilization and ROS production. This leads to the release of lysosomal contents such as cysteine protease cathepsin B, which is detected by the nod-like receptor (NLR) family Pyrin domain containing 3 (NLRP3). Following this, the activation and subsequent formation of the inflammasome complex leads to the production of interleukins and activate immune cells [74].

The SARS-CoV-2 receptor-binding domain (RBD) on S protein binds strongly to human and bat angiotensin-converting enzyme 2 (ACE2) receptors, resulting in specific humoral responses through RBD-specific antibodies secretion neutralization. This method has an exciting potential for use in developing RBD-based vaccines against SARS-CoV-2 infections [91]. Along with this approach, immunogenic epitopes of the spike glycoprotein with antigenic properties were also characterized by immunoinformatic analysis and their promising capacity to formulate a multi-epitope peptide vaccine [92].

In general, there are two types of vaccination methods in VARID: Systemic vaccination and intranasal vaccination. Intravenous administration is used for some NP-based drugs, but is not considered an optimal route for vaccination. In systemic vaccination, the final formulation of vaccine, which can be mRNA, recombinant protein, attenuated virus, or virus-like particle, is injected deep into the deltoid muscle. Vaccine particles are taken up by tissue resident APCs (macrophages and dendritic cells) and are transported via the lymphatic system. Once in the lymph nodes, these antigens are presented to T cells to induce the specific plasma cells differentiation of Type 1 helper T cells and CTLs. Once activated plasma cells produce IgG against viral antigens, and CTLs kill virus-infected cells. Circulation of the vaccine particles in the lymphatic vessels can transport it to the BALT, where it also helps to generate a specific local antiviral response. However, the structure of the vaccine is an important consideration for stability and sustained antigenic release applications; this is referred to as the depot effect.

Alternatively, in intranasal vaccination, stable vaccines are formulated, such as mRNA loaded LNPs and are introduced to the nasal cavity through swaps or sprays. They are then absorbed into the mucosa, where the particles penetrate into the epithelial layer and are taken up by M cells. In the secondary lymphatic associated tissues in the lamina-propria, the vaccines antigens are presented to T cells, causing plasma cell activation, leading to IgA production. Dimeric secretory IgA block the virus and prevent infection [93].

Compared to conventional approaches, recent advances in vaccine nanomedicine offer superior therapeutic potential for viral respiratory diseases [94]. The unique features of the nanoparticles, including small particle size (100–200 nm), adjustable surface charge, and specific surfaces, result in a powerful platform for pharmaceutical applications and medicine. In recent years, many nanoparticles’ formulations, including liposomes, polymers and dendrimers, inorganic nanoparticles (silver nanoparticles, gold nanoparticles), mesoporous silicon nanoparticles, and quantum dots, have been developed to meet the challenges against viral infections. In this section, the importance of nanoparticle-based systemic therapeutic and vaccine administration for targeting immune system cells and BALT, suppressing hyper inflammation, and targeting various viral structural proteins, are summarized.

### 3.1. Nanocarriers for Targeted Anti-Viral Drug Delivery and Nano-Vaccine Design

#### 3.1.1. Liposomes

Liposomes are spherical shape lipid nanoparticles (20–200 nm) composed of a synthetic or natural phospholipid bilayer. Liposomes mimic cell membrane’s structure and can carry both hydrophilic and lipophilic pharmaceutics compounds [95]. The size and surface charge diversity make liposomes good candidates for non-cytotoxic and biodegradable drug delivery carriers, especially as an adjuvant in vaccine delivery studies [96,97]. The vesicle size of the liposomes for drug and vaccine delivery can have a significant role in triggering the immune activation process as soon as introducing to the body through the development of different pathways, such as Th1- or Th2 responses [98]. Additionally, they can stimulate the APCs uptake rate based on their size and surface charge. The surface charge of the liposomes can also have a giant effect on the rate of Ag loading efficacy through either entrapment or electrostatic adsorption methods [99].

RES has a potency for entrapment by liposomes, making them suitable for targeted delivery in APCs. This mechanism is the basis of vaccine design [100]. According to this phenomenon, many types of vaccines have been designed against SARS-CoV-2. Lipid nanoparticles (LNP) are considered revolutionary for vaccination development, with BNT162b2 (Pfizer^®^) being the first FDA approved LNP vaccine [101]. Here, the mRNA-based nano-vaccine contains a full-length mRNA spike protein of SARS-CoV-2 in an LNP composed of ((4-hydroxybutyl) azanediyl) bis (hexane-6,1-diyl) bis(2-hexyldecanoate), 2 [(polyethylene glycol)-2000]-N, N-ditetradecylacetamide, 1,2-distearoyl-sn-glycero-3- phosphocholine, and cholesterol), potassium chloride, monobasic potassium phosphate, sodium chloride, dibasic sodium phosphate dihydrate, and sucrose. A second’s mRNA-based SARS-SoV-2 vaccine is Moderna^®^, which is comprised from mRNA1273 encoding full length spike protein loaded on an ionic LNP (SM-102) containing dimyristoyl glycerol (DMG) as an artificial phospholipid, cholesterol, and a modified polymer of ethylene glycol (Polyethylene glycol (PEG) 2000), 1,2-distearoyl-sn-glycero-3-phosphocholine [DSPC]), sucrose, acetic acid, sodium acetate and tromethamin hydrochloride [102]. In addition to the slight differences in mRNA sequence, the ratio of nanoparticle compounds in these two vaccine formulations also have special differences. The Moderna^®^ vaccine does not have restrictions on transport and storage at ultra-cold temperatures and it is advantageous for nanotechnology. Finally, ARCoV is a thermostable mRNA vaccine that encode receptor binding domain (RBD) of SARS-COV2 and is encapsulated in an LNP [103].

#### 3.1.2. Polymeric Nanoparticles

Polymeric nanoparticles (PNs) are another example of nanocarriers for anti-viral therapeutic delivery systems. PNs are generally composed of monomeric units in the form of a colloidal phase, categorized into synthetic polymers, natural polymers, and copolymers. Besides the application of polymeric nanostructures in the pharmaceutical industry, PNs have multiple advantages for vaccine delivery applications, including controlled release of antigens, intracellular persistence in APCs, and adjustable properties such as size, composition, and surface properties [104]. Although PNs can inhibit the cytotoxicity of the common molecular adjuvants by encapsulating them, the potential toxicity of the polymeric nanoparticle by itself is another significant issue that should be considered in using them as a vaccine delivery system [105]. This might arise from some surfactants which are used during the chemical synthesis of the nanoparticles [106]. Moreover, probable aggregation of some nanoparticles after Ag loading might be also effective in inducing cytotoxic response in-vitro or in-vivo [107]. PN size and surface charge have an important role in passive diffusion of them towards the lymph nodes and their interaction with APCs such as internalization by DCs or macrophages [105]. Similar to LNs, the surface charge of the PNs shows a great effect on the activation of the T helper cell types so that cationic PNs trigger the Th1-based immune responses, while anionic ones can activate both Th1 and Th2 immune responses [108].

Commonly used polymeric nanoparticles for such applications are PLGA, poly-ε caprolactone (PCL), poly-(γ-glutamic acid) (γ-PGA), polymethylmethacrylate (PMMA), poly-alkyl-cyanoacrylates, polyvinylpyridine, polygluteraldehyde, polyacrylamides, polyethyleneimine (PEI), gelatin chitosan, and human serum albumin (HSA) [109]. Similar to liposomes, PNs are quickly taken up by the RES and Kupffer cells, with similar effects [110]. In one study, a poly (ethylene oxide)-modified poly (ε-caprolactone) (PEO-PCL) nanoparticulate system was developed for the encapsulation of saquinavir (SQV), an antiretroviral agent, using a solvent displacement process. THP-1 cells of the monocyte/macrophage origin demonstrated rapid cellular uptake of the encapsulated PEO-PCL nanoparticles. Intracellular SQV concentrations of the PEO-PCL-SQV nanoparticles were significantly higher than that of aqueous SQV solutions, indicating their benefits in viral therapy [111]. As previously mentioned, modified PEG is used in the final compound of both Pfizer^®^ and Moderna^®^ mRNA-based vaccines. NVX-CoV2373 (Novavax) is a protein-based vaccine containing saponin Matrix-M™ adjuvant [112]. With the use of polymeric nanoparticles, drug molecules are protected and both therapy and imaging can be combined [113,114]. Other promising characteristics of the polymeric nanoparticles such as biocompatibility [115], long-time spatiotemporal stability [116,117], and pathogen-like characteristics [118] make them a suitable candidate for intranasal vaccine administration [119].

As an example, PLGA nanocapsules have been used for the encapsulation of various virus antigens or RNA for systemic or local nano-vaccine designing. The safety of poly lactic-co-glycolic acid (PLGA) as a biodegradable and biocompatible polymer has been approved by the FDA and European Medicines Agency (EMA) for medicinal applications [120]. The encapsulation of different biomolecules in PLGA nanoparticles can result in sustainable release over long time periods [121,122], which is a critical result of mucosal vaccinations [123]. For instance, PLGA nanoparticles have been used for encapsulation of the bovine parainfluenza 3 virus (BPI3V) antigen as a novel intranasal nano-pharmaceutical vaccine. The immune response against BPI3V was compared to the current commercial version used in dairy calves. Following nanoparticle vaccine administration, the early induced immune response demonstrated continued growth until the end of the study, while similar results were not observed in the bare (BPI3V) antigen vaccine groups [123]. The spherical PLGA-NPs (200–300 nm) encapsulated inactivated swine influenza virus (SIV) H1N2 antigen (KAg) via water/oil/water double emulsion solvent evaporation. The pigs were infected with a virulent heterologous influenza virus strain after double vaccination with this preparation. The animals showed significantly milder disease, reduced lung pathogenicity, and substantial clearance of the virus from the lungs compared to the non-vaccinated control group [124].

In another study, Okamoto and his co-workers designed a nanoparticle by adding γ-PGA hydrophobic derivative (a natural polymer) to influenza virus hemagglutinin (HA) vaccine, in order to amplify the protective immune responses against influenza virus (H1N1) in the mice. The researchers proved that the PGA-NP HA vaccine was more effective in protection against different influenza viruses when administered via intranasal vaccination, compared to subcutaneous injection [125].

Chitosan, a natural polymer composed of randomly distributed β-(1–4)-linked d-glucosamine, N-acetyl-d-glucosamine, and N-(2-hydroxypropyl) methacrylamide/N-isopropylacrylamide (HPMA/NIPAM), are two frequently used polymeric nanoparticles for nano-vaccine delivery. Chitosan nanogels have been investigated as intranasal vaccine nanocarrier against different respiratory viruses, specifically influenza virus, with enhanced mucosal and systemic immune responses demonstrated in pigs [125,126]. Chitosan nanogels have also shown high levels of systemic and mucosal antibodies, as well as serum HI titers, in mice [127]. Another polymeric nanocarrier, polyanhydride, has been used to encapsulate the post-fusion F and G glycoproteins from bovine RSV. This formulation showed increased mucosal and systemic antiviral immunity in a neonatal calf model, which can potentially be generalized to humans [128].

#### 3.1.3. Dendrimers

Dendrimers are a group of star-shaped three-dimensional macromolecular networks with some particular properties that make them a lucrative nanocarrier for anti-viral therapy. It is theorized that their anti-viral effect mechanism is attributable to host cell-virus interaction disruption during infection, where they form stable complexes with viral structures or receptors at the surface of cells [125]. In this regard, scientists evaluated the anti-viral capacity of 3′-sialyllactose- and 6′-sialyllactose-conjugated dendritic polymers against human and avian influenza viruses. The disaccharide lactose and the tri-saccharides 3SL and 6SL conjugate to primary amines in polyamidoamine (PAMAM) dendrimer backbones for the generation of 4 and 8. The results demonstrate that octavalent compounds are more potent than the tetravalent. Furthermore, human IAV strains can be subdued by (6SL) and, to a lesser extent, by (3SL)-conjugated PAMAM dendrimers [129]. Researchers also developed anionic and cationic polyamidoamine with PAMAM on MERS-COV infectious, with their intervention showing improved antiviral responses [130]. KK-46 dendrimer is a peptide-based compound used for intracellular delivery of anti-SARS-CoV-2 siRNA for inhibition of virus replication [131]. Finally, astrodimer sodium is a four-lysine dendrimer with a poly anionic charge that has been shown to inhibit viral infections in VeroE6 cells and reduce the replication of virus [132].

#### 3.1.4. Quantum Dots and Inorganic Nanoparticle

Quantum dots (QDs) are semiconductor inorganic nanocrystals with size-dependent optical and electronic properties, and have been widely used for virus detection and imaging, given their inherent fluorescent emission [133]. QDs can also be used in viral replication inhibition approaches due to their inherent additional anti-viral capabilities. To this end, Huang et al. formulated benzoxazine monomer derived carbon dots (BZM-CDs) and demonstrated their infection-mitigation ability against flaviviruses and non-enveloped viruses, such as adenovirus-associated viruses. It was found that the viricidal ability of functional BZM-CDs was related to surface binding of virions, which inhibited the main step of virus and host cell interaction [134]. The same group has also found that carbon quantum dots (CQDs) with subtle ligand modifications can play an outstanding role in inhibitory activities against human coronavirus [135]. 4-aminophenyl boronic acid hydrochloride (4-AB/C-dots) is another QD compound with very powerful antiviral effects, especially in HSV [136]. In addition to the success of these QDs in the fabrication and design of the SARS-CoV diagnostic aptamer-based chip at 2011 [137], these elements can be used to carry nucleic acid-based antigens, such as dsDNA (viral vector) and mRNA vaccines.

Other inorganic nanoparticles include mesoporous silica as well as metal oxide NPs like zirconia (ZrO_2_) NPs, zinc oxide nanoparticles, titanium dioxide (TiO_2_) NPs, silver NPS (Ag-NP), and gold NPs (Au-NP) [138]. Among these options, mesoporous silica (MSN) has been widely utilized for anti-viral applications. Antiviral drugs are known to be localized into their target location based on blood circulation, which can cause substantial side effects. To alleviate this issue, mesoporous silica nanoparticles (SiNPs) are considered an ideal anti-viral drug delivery nanocarrier for targeting specific viruses through binding to the viral proteins. Consequently, due to the unique porous structure, SiNPs can inhibit infectious virus strain replication through either their antiviral characteristics or by providing a sustained release profile of the antiviral pharmaceutic agents. Therefore, one of the features of SiNPs is their significant inherent antiviral agent without any selective vaccines or specific drugs for treatment [139]. Karamov et al. have evaluated the antiviral activity of silica nanoparticles against respiratory syncytial virus (RSV) and determined their high viricidal capacity. The researchers demonstrated that non-specific interactions between SiNPs and viruses resulted in the blocking of virions by SiNPs [140]. In order to study the anti-viral properties of the other inorganic nanoparticles, Huo et al. evaluated the antiviral effect of ZnO_2_ nanoparticles against avian influenza virus [141], with the aim of proving the protecting activity of zirconia NPs against the highly pathogenic virus without side effects. In this regard, zinc oxide nanoparticles (ZnO-NPs) were also designed for the inhibition of H1N1 influenza virus. It was shown that PEGylated ZnO-NPs were antiviral agents, effective in countering H1N1 influenza virus infections [142]. Additionally, the anti-viral properties of titanium dioxide (TiO2) nanoparticles were assessed, and it was confirmed that they could potentially inactivate the influenza virus H3N2 by directly destroying the virus particles [143].

While gold nanoparticles are most commonly used for rapid SARS-CoV-2 diagnostic kits [144,145], the presence of these nanoparticles in the final composition of vaccine enhances the adjuvant performance and immune response. Au NPs can be used in intranasal vaccines and can infiltrate into the lymph nodes, where they trigger a significant antigen-specific cytotoxic T cell immune response [146]. Tao W et al. demonstrated that Au NPs formed a new complex with non-native cysteine residue at the C-terminal of influenza M2 via thiolate group ((M2) e-Au NP). The complex was intranasal administered to mice along with cytosine-guanine rich oligonucleotide (CpG) adjuvant, which triggered a protective immune response against PR8 influenza A virus [147]. This formulation also showed protection against *A/California/04/2009* (H1N1pdm) pandemic strain, A/Victoria/3/75 (H3N2) strain, and *A/Vietnam/1203/2004* (H5N1) infections [89]. Silver nanoparticles (AgNPs) have also been used in formulations with the plasmid-encoding hemagglutinin (HA) gene of avian influenza virus *A/Ck/Malaysia/5858/04* (H5N1) (pcDNA3.1/H5), and were shown to induce both antibody and cell-mediated immune responses with enhanced cytokine production [148]. Hanako Sekimukai et al. produced a nano-vaccine of Au NP-coated spike protein and two TLR agonists (LPS and poly:IC) as adjuvants. The results of this study showed that this vaccine induces a strong antigen-specific IgG response against SARS-CoV-2, but it is not protective enough to inhibit eosinophils chemotaxis in the lungs [149].

#### 3.1.5. SAPNs and VLPs

Self-assembling protein and peptide nanoparticles (SAPNs) are complexes made from monomeric protein oligomerization using recombinant technologies and are considered suitable candidates for pharmaceutical nanocarriers [150]. They can be formed in nano-diameter ranges and used as nano-vaccine candidates against viruses, making them suitable for intranasal delivery [150,151]. They can be designed to mimic viruses or bacteria in size and surface antigenicity and have been reported to elicit CD8^+^ T cell responses.

In one study, SAPNs were used against the purified coronavirus spike protein in the Middle East Respiratory Syndrome coronavirus (MERS-CoV) and ferritin [152,153]. In another study, assemblies of four tandem copies of M2e and headless HA proteins of influenza virus stabilized by sulfosuccinimidyl, were prepared. Vaccinations with these nanoparticles in mice induced robust, long-lasting immunity with complete protection against challenging symptoms induced by divergent influenza A viruses [154].

Further, Linling He et al. decorated a three-part SAPN including RBD, the modified spike peptide (S2G-HR2) of SARS-CoV-2, and ferritin, using a SpyTag/SpyCatcher system. This formulation enhanced the neutralizing antibody of SARS-CoV-2 ten times more than one SANP and other nanoparticles [155]. Kang et al. worked on SANP-based vaccines using two-part SAPN with modified RBD (mi3), ferritin, and RBD-153-50NP with the SpyCatcher system, and individual SANPs. Their interventions on animal models (BALB/c) showed improved thermostability for RBD-153-50NP, compared to other NPs. In fact, the antibody titer of this compound was much better than other compounds [156]. VLPs are another type of nano-vaccines that mimic the structure and the antigenic epitopes of their virus without including genetic material. They also promote efficient phagocytosis by APCs and immune response activation [157,158,159]. Today, ‘smart’ VLPs are often created using immunoinformatic strategies, the identification of epitopes, and artificially and genetically modifications. Construct design and viral vector engineering usually plays a very important role in this regard. Combining the VLPs with other nanoparticles is the basis of an effective vaccine [160]. It has also been reported that intranasal delivery of VLPs composed of 5 repeats of *M2e* epitopes (*M2e5x*) of the influenza virus resulted in strong humoral and cellular immune responses, therefore providing protection against different serotypes of influenza viruses [161]. In another effort, self-assembling repeats of the severe acute respiratory syndrome (SARS) B cell epitope from the C-terminal heptad of the virus’ spike (S) protein resulted in VLPs with the size of 25 nm. It showed the significant antibody response specific for the coiled-coil epitope of the peptide [123]. Xu et al. designed a four gene (M, N, S, and E) construct in a pcDNA3.1 mammalian expression vector and harvested VLP from the resulting supernatant [162]. Similarly, Swann et al. used CMV-driven mammalian expression vectors for three SARS-CoV-2 genes (E, M and S) and harvested the VLPs from HEKT293 cell supernatant (154). Further, Lu et al. combined an mRNA vaccine against RBD (*RQ3011-RBD*) with three structure VLP (S, E, and M) and LNPs for comparison. Notably, the NAb titration of mice that received RQ3013-VLP was significantly higher than the S-specific binding antibody of mice that receiving RQ3012-spike [163]. A summary of nanoparticle-based vaccine formulations that have been used against respiratory virus infections are shown in Table 2.

## 4. Local Airway Delivery of Nanoparticles in VARID

In addition to systemic nano-vaccine delivery, therapeutic medications can be directly delivered to the lungs via intranasal administration. This method of airway delivery provides advantages over systemic methods such as targeted delivery, rapid drug absorption due to the high surface area of the capillary network and extensive vascularization of lungs, protection of drug molecules from enzymatic degradation due to fewer degradative enzymes within the lungs, and most importantly, minimally invasive delivery [178,179]. The nano-formulations of drugs may improve the delivery rate into the lungs either as a colloidal dispersion in a medium using nebulization or as a dried powder using pressurized metered-dose inhalers and dry powder inhalers [180]. Dry powders are typically preferred given the benefits of longer shelf-life, stability, simple administration, and better aerodynamic properties [181]. The methods of synthesis mainly include co-precipitation, nanoprecipitation, spray-drying, freeze-drying, and microemulsion [182,183,184,185,186].

### 4.1. Intranasal Airway Delivery of Therapeutic Nano-Carriers in VARID

Nanotechnology can potentially facilitate the efficacy of advanced therapeutics or vaccines by encapsulation inside the micro/nano-carriers to be administered using intranasal inhalation, as opposed to systematic delivery. In this way, Broichsitter et al. claimed that the anti-inflammatory corticosteroid Salbutamol could be effectively loaded in a polymeric nanocarrier composed of poly (vinyl sulfonate-co-vinyl alcohol)- graft-poly (D, L-lactide-co-glycolide, PLGA) for sustained pulmonary drug release [187]. To further enhance the selectivity of vaccine/drug delivery, the nanocarrier can be designed to have a targeted and smart release approach through stimuli-responsive delivery systems. As an example, the anti-inflammatory therapeutic hydroxy benzyl alcohol was incorporated into polyoxalate, which responds to hydrogen peroxide. The drug incorporated polymer was then encapsulated inside PLGA nanoparticles. The results showed that the cleavage of peroxalate ester links between the drug and the polyoxalate polymer in the presence of hydrogen peroxide releases the drug to improve selectivity and environmental responsivity in drug delivery [188,189].

Other specific drugs, such as antibiotics can also be encapsulated inside nano-carriers to enhance the efficacy of therapy against bacterial lung infections through intranasal administration [190,191]. In the same way, the co-encapsulation of multiple antimicrobial agents can potentially improve the efficacy of the VARID treatment process [191]. For example, Quercetin, an antioxidant and anti-inflammatory drug, was encapsulated inside solid lipid nano-carriers for airway delivery to the lungs via nebulization [192]. The results of this study clarified the capability of the nano-carrier use for localized delivery and deposition inside deep lung areas [192]. Among the various nanocarriers, liposomes have attracted ample attention for airway therapeutic delivery to the lung. Inhaled liposomal formulations for localized airway delivery has been widely evaluated for the treatment of lung infection diseases. Currently, there exist commercially available liposome inhalation products such as Arikayce^®^ (amikacin, Insmed, Monmouth Junction, NJ, USA) liposome inhalation suspension. In a related study, a liposomal formulation of ciprofloxacin with the average size of 350 nm was synthesized using a film-hydration method with phospholipids and cholesterol as precursor materials. Besides the high drug loading efficiency (ca. 93%), the amount of drug release from the liposomal formulation was shown to be higher in the simulated lung medium compared to the saline medium, thus, demonstrating selective drug release in a lung simulating medium. Furthermore, in vivo studies in rat models revealed the effective lung targeting ability of the drug-loaded liposome formulation in comparison to the free form of the drug [193].

Airway delivery of therapeutic nucleic acids (DNA and RNA) is also a practical approach for the treatment of side effect diseases caused by a viral infection. The invasion of viruses causes several lung associated diseases through their pathogenic genes infection or genetic malfunction [194]. Therefore, gene therapy is a practical approach against certain viral infections. It was reported that the delivery of mostly negatively charged nucleic acids is difficult when they are in a free form [195]. Subsequently, positively charged polymers such as chitosan and polyethyleneimine can be utilized for the encapsulation of negatively charged nucleic acids with the aid of electrostatic attraction forces.

Airway delivery of drug-loaded nano-carriers to the lungs and other parts of the respiratory tract could be performed by various methods such as nasal or oral spray, nebulization, dry powder inhaler devices or pressurized metered-dose inhalers [196]. Among these methods, inhaler devices provide some advantages over nebulization, such as higher efficiency, higher portability, no propellant requirements, simplicity in use, and longer shelf-life. However, inhaler devices for gene delivery suffer from some disadvantages such as difficulty in controlling the flow rate, dose uniformity, and reduced control of environmental effects on formulation integrity. To address these problems, several studies have been conducted on gene delivery using inhalable micro/nanocarriers systems. In such a study, a pressurized metered-dose inhaler was utilized for airway delivery of DNA envelope in which surfactant-coated DNA particles were prepared using the reverse microemulsion method [197]. In another study, the complex coacervation encapsulation method was utilized for the preparation of a chitosan nano-carrier loaded with DNA. Chitosan nanocarriers were later coated with a water-soluble and biodegradable layer of a hydrofluoroalkane [198]. The results showed that this design could be utilized for gene delivery to the lungs using pressurized metered-dose inhaler devices at a relatively low cost with high portability for the potential treatment of VARID diseases such as asthma and COPD. Further, small interfering RNAs (siRNA) are a relatively new class of therapeutic agents for the treatment of lung diseases through controlled levels of transcript at specific molecular targets. Therapeutic siRNAs could be similarly conjugated with positively charged polymers such as chitosan and polyethyleneimine and delivered locally in the deep lung area. In this regard, other nonviral polycationic polyplexes have been developed, and their efficiency for siRNAs delivery to lungs in mice models was evaluated [199,200]). However, the delivery of these polycationic polyplexes or free siRNA faces drawbacks such as the possibility of the aerosol degradation by the shear stresses caused by nebulizer devices. To address this issue and improve the efficacy of delivery, encapsulation of siRNAs inside biodegradable and muco-adhesive nanocarriers such as chitosan and PLGA, with high loading efficiencies and high stability in airways and lung mediums, have been tested. Nano-carriers composed of PLGA for carrying siRNA provides a sustained-release profile and higher colloidal stabilities compared to polycationic polyplexes, and are favorable for siRNA local delivery.

In the case of SARS-CoV-2 as a type of VARID, various FDA-approved prescribed drugs have been evaluated for the treatment of infected patients [201,202,203,204]. However, despite considerable nanotechnology research and patent publications on various aspects of the coronavirus treatments [205,206], there is only one report on the utilization of nanotechnology-based design to address SARS-CoV-2 VARID using a localized airway delivery route. This nano-formulation of pharmaceutics design suggests the application of a previously developed nano-carrier based on chitosan (Novochizol™) for delivery of potential anti-COVID-19 drugs to the lungs [207]. Therefore, there is a remarkable capacity for the development of new drug formulations to prevent and/or treat the newly emerged SARS-CoV-2 virus, using nanotechnology enhanced airway delivery drugs through modulation of molecular targets or treatments of VARID. Aerosol liposomal therapy has also been used for several years with acceptable and safe clinical results [122,125], in terms of potential SARS-CoV-2 infection prevention and treatment, some reports claimed the efficiency of inhalation and oral use of a liposomal formulation of lactoferrin [124].

### 4.2. Intranasal Airway Delivery of Nano-Vaccines in VARID

Intranasal vaccines, in addition to antigen delivery to the epithelial cells and local APCs at the site of infection, also stimulate NALT and BALT to produce IgA, which blocks the binding and entry of the virus. The formulation of these types of vaccines is a key point in the design and manufacturing of vaccines with enhanced stability and efficacy. These vaccines may be used in single doses due to the strong enrollment of mucosal immunity [58]. ChAd intranasal vaccine it is formulated with polymeric NPs is a novel inhaled nasal spray vaccine for inducing powerful mucosal protection against SARS-CoV-2 after just a single dosage [164]. Examples of intranasal vaccines against VARID are summarized in Table 3.

**Figure 3:**
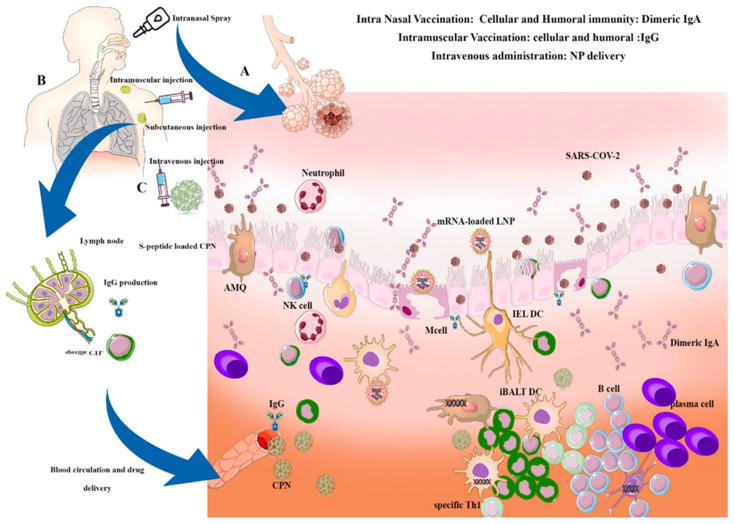
Mechanisms of vaccine administration using nanoparticles in VARID. (**A**) Intranasal vaccination: The aerosol-based nanoparticles containing the mRNA of virus antigen is transferred through the mucus layer into the nasal epithelial tissues by micro-fold cells (M cells) or passively through epithelial cell junctions. Nanoparticles are captured by DCs, and alveolar macrophages (AMQ) are passed by epithelial junctions and by other APCs, such as B cells. The mRNA of the antigen is translated into a specific peptide and presented to immature T cells, activating them and B cells. The activated B cells proliferate in the B cell zone to maturity and enter the systemic circulation to reach the inflammation site. IgA and B cells locally differentiate into antibody-secreting plasma cells to produce IgA dimers. The IgA dimers are secreted via polymeric Ig receptor (pIgR) at the mucosal surface. NALT/BALT immune response induces long-lasting B and T memory cells able to activate a rapid memory response [110]. (**B**) Other types of nano-vaccine injection, such as intramuscular, subcutaneous, and intravenous, can induce systemic reactions and IgG production, thus inducing lung protection. (**C**) Some specific nanoparticles induce the immunomodulatory responses using CPN, which can induce the IgG and specific CTL production against antigens. Systemic injection of nanoparticles can induce iBALT and local responses.

Figure 3 illustrates the schematic view of different routes of nano-vaccine administration in respiratory viral infections such as SARS-COv-2. Different nanoparticles containing genetic materials of the virus interact with different immune cells through NALT/BALT immune responses.

Localized delivery of the antigens and epitopes to the specific cells in the respiratory tract requires the rational design of nano-carriers for targeted delivery in a controlled and prolonged manner [84,85]. Nevertheless, some reports indicate that certain nanoparticles have intrinsic immunomodulatory activity; these include carbon nanotubes, gold, TiO_2_, and SiO_2_ nanoparticles [210,211]. Antigens can be loaded using different approaches such as physical encapsulation or chemical bond conjugation. For the rational design of localized administered nano-vaccine for the management of respiratory diseases, the following criteria should be considered. Firstly, the nano-vaccine size should be similar to the target viruses (i.e., 20–200 nm), in order to pass over the biological barriers of the respiratory tract. Therefore, the nano-vaccine size distribution should be carefully adjusted. Secondly, the nano-vaccine should be positively charged to induce stronger immune responses in comparison to the negatively charged nanocarriers [212,213]. Next, the nano-vaccine should mimic the virus structure, allowing the antigens/epitopes to be effectively encapsulated inside the nano-carrier or immobilized on the nanoparticle surface. In addition, the formulation of the nano-vaccine and its route of administration should be carefully evaluated. As per previous studies, it has been reported that airway vaccination through mucosal administration via oral or intranasal routes provide more efficiency in the management of respiratory viral infections so that strong cellular and humoral immune responses are induced systemically or at the mucosal surfaces [214,215]. The intranasal route is preferred as it leads to higher antigen-specific lymphocyte proliferation, induction of antigen-specific IgA antibodies, and cytokine production [216,217,218,219,220]. Last, the nano-carrier should not induce any significant cytotoxicity to non-targeted cells. As such, the carrier material should be biocompatible and biodegradable [201,221].

## 5. Key Points on Nanomedicine and Nano-Vaccine against COVID-19

COVID-19 vaccine candidates may follow different technology platforms [201,220]. Live attenuated vaccines use live virus with reduced virulence, which can induce a strong immune response, but may be dangerous for immunosuppressed individuals. Despite a 100-year history of producing vaccines against viral infections using the traditional attenuated live attenuation method, the first vaccines to receive emergency approval for general use in the United States and other countries were new generations of vaccines, including mRNA and vector-based vaccines [101]. Viral-vector based vaccines are among the other type of vaccines using a non-pathogenic viral backbone, such as adenovirus (ChAd, Ad5, Ad26) to introduce a SARS-CoV-2 gene, such as S, RBD, E, and M, into the host cells. A preliminary report of purified inactivated vaccine candidate (PiCoVacc) against SARS-CoV-2 demonstrated complete protection in non-human primates against SARS-CoV-2 strains by eliciting the potent humoral response of SARS-CoV-2-specific neutralizing antibodies. Another study based on the S1 protein nano-vaccines has received FDA approval for emergency vaccination (Novavax, NVX-CoV2373). This vaccine is composed of VLP nanocarriers containing recombinant spike protein and the suponin-based adjuvant Matrix-M. This technology follows the researcher’s patented nano-formulation WO2015042373, in which VLPs containing at least one trimer of S protein induced neutralizing antibody response in mice and transgenic cattle [221].

Despite the overall success of mRNA vaccines in inducing a proper immune response, nano-formulation of these subcategories via encapsulation in positively charged nanocarriers, can potentially enhance the stability and reduce the delivery challenges of mRNA or DNA across the cell membrane or even through the cell nucleus. In this regard, the patent application (WO2017070626) by Moderna Inc. disclosed mRNA vaccines consist of mRNAs encoding viral antigenic full-length S, S1, or S2 subunits from SARS-CoV and MERS-CoV viruses, formulated in cationic lipid nanoparticles. The mice vaccinated with mRNA encoding full-length S protein elicited much higher neutralizing antibody titers when compared to the S2 subunit. In this study, New Zealand white rabbits immunized with the MERS-CoV mRNA vaccine, encoding the full-length S protein, demonstrated a 90% reduction in viral load with significant levels of neutralizing antibody against MERS-CoV. On February 2020, Moderna announced the release of the first batch of mRNA-1273 against SARS-CoV-2 for use in humans.

The most advanced candidates that have recently shifted into clinical development include mRNA-1273 from Moderna (LNP-encapsulated mRNA vaccine encoding S protein), Ad5-nCoV from CanSino Biologicals (Adenovirus type 5 vector that expresses S protein), INO-4800 from Inovio (DNA plasmid encoding S protein delivered by electroporation), and LV-SMENP-DC (DCs modified with a lentiviral vector expressing synthetic minigene based on domains of selected viral proteins; administered with antigen-specific CTLs), and pathogen-specific APC (artificial APCs modified with a lentiviral vector expressing synthetic minigene based on domains of selected viral proteins) from Shenzhen Geno-Immune Medical Institute. The clinical trial details of these candidates are summarized in Table 4 [222].

## 6. Conclusions and Future Perspectives

Engineered nanocarriers have demonstrated their outstanding role in efficient drug and vaccine delivery against viral diseases. Various nanostructures have been proposed for use as carriers for antiviral deposition. Depending on the target tissue, these structures can be chemically modified to enhance conventional antiviral properties by providing controlled release and drug protection with the aid of nano systems. In the case of vaccine production, the use of nano-formulations as carriers of adjuvant therapies can further improve the shortcomings of conventional vaccines by enhancing antigen stability and targeted delivery properties. In relation to the specific nano-vaccines used for immunizing against respiratory viruses, local administration through the nasal route generates efficient mucosal or systemic immunity. The immunogenicity of these nano-formulated vaccines can be enhanced through facile recognition and endocytosis by the APCs.

As a perspective view of the vaccines and nano-vaccines development against the COVID-19 pandemic, the structural proteins of SARS-CoV-2 make attractive candidates and should be considered as necessary elements for the cellular infection and virion assembly [223]. As the global population faces compounding challenges involving the COVID-19 pandemic, an effective vaccine plays an undeniably important role in controlling the spread of SARS-CoV-2. However, more comprehensive information is needed about the virus, as well as around the specific immune response that it elicits in the human body.

## Figures and Tables

**Table 1:** Common viral respiratory infections and the associated respiratory infection disease.

Virus	VARID	Ref
Adenoviruses	Common Cold, Pneumonia	[15]
Coronaviruses	Common Cold, SARS, MERS, COVID-19	[6]
Enteroviruses	Common Cold	[16]
Influenza Virus (Types A and B)	Influenza, Pneumonia	[17]
Metapneumovirus	Common Cold, Pneumonia, Bronchiolitis	[18]
Parainfluenza Virus (Type 3)	Common Cold, Croup, Pneumonia, Bronchiolitis	[19]
Parainfluenza Viruses (Types 1, 2)	Croup	[19]
Respiratory Syncytial viruses	Pneumonia, Bronchiolitis	[20]
Rhinoviruses	Common Cold	[21]

**Table 2:** Nanoparticle-based vaccine formulations against respiratory virus infections.

Nanoparticle	Size (nm)	Virus	Antigen/Epitope	Adjuvant	Status	Route of Administration	Ref
INORGANIC NANOPARTICLES							
Gold	12 Influenza		M2e	CpG	Preclinical	IN	[88]
	173 ± 2.4	SARS-COV 2	S	LPS, P:IC	Preclinical	SC	[145]
POLYMERIC NANOPARTICLES							
PLGA	225.4	Bovine parainfluenza 3 virus	BPI3V proteins	-	Preclinical	IN	[121]
	200–300	Swine influenza virus (H1N2)	Inactivated virus H1N2 antigen	-	Preclinical	IN	
γ-PGA (Poly-glutamic acid)	100–200	Influenza (H1N1)	HA	-	Preclinical	IN	[123]
Chitosan	140	Influenza (H1N1)	H1N1 antigen	-	Preclinical	IN	[125]
	300–350	Influenza (H1N1)	HA-Split	-	Preclinical	IM	[162]
	571.7	Swine influenza virus (H1N2)	Killed swine influenza antigen	-	Preclinical	IN	[124]
	200–250	Influenza (H1N1)	M2e	Heat shock protein 70C	Preclinical	IN	[164]
	125	SARS-COV2	S	-	Preclinical	IN	[163]
HPMA/NIPAM	12–25	RSV	F protein	TLR-7/8 agonist	Preclinical	IN	[165]
Polyanhydride	200–800	RSV	F and G glycoproteins	-	Preclinical	IM	[126]
SELF-ASSEMBLING PROTEINS AND PEPTIDE-BASED NANOPARTICLES							
N nucleocapside protein of RSV	15	RSV	RSV phosphoprotein	R192G	Preclinical	IM	[166]
	15	RSV	FsII	Montanide^TM^ Gel 01	Phase I	IM	[167]
	15	Influenza (H1N1)	M2e	Montanide^TM^ Gel 01	Phase I,II	IN	[168]
Ferritin	12.5	Influenza (H1N1)	M2e	-	PhaseII	IN	[169]
Q11	-	Influenza (H1N1)	Acid polymerase	-	PhaseI,II	IN	[169]
S2G-HR2-RBD		SARS-COV 2	RBD-S		PhaseII	IM	[151]
RBD-153-50	50.67±0.11	SARS-COV 2	RBD		PhaseII	IM	[152]
LuS *-S-F **	50	SARS-COV 2	S-LuS-F	SAS ***	PhaseII	IN	[170]
OTHERS							
VLP	80–120	Influenza (H1N1)	HA	-	Preclinical	IM	[171]
	80–120	Influenza (H1N1, H3N2, H5N1	M2e	-	Preclinical	IM	[159]
	80–120	RSV	F protein and G glycoprotein of RSV and M1 protein of Influenza	-	Preclinical	IM	[172]
	100	SARS-COV 2	M-N-S-E	-	Preclinical	IN	[158]
	100	SARS-COV 2	M-S-E	-	Preclinical	IM	[160]
ISCOM (Quillaia saponin, cholesterol, phospholipid, and associated antigen)	40	Influenza (H1N1)	HA	ISCOMATRIX	Preclinical	IN	[173,174]
DLPC liposomes (Dilauroylphosphatidylcholine)	30–100	Influenza (H1N1)	M2, HA, NP	MPL and trehalose 6,6′ dimycolate	Preclinical	IN	[175]
Surface-linked liposomal peptide	-	Vaccinia virus	SARS-CoV N epitopes	-	Preclinical	IM	[176]
Cationic lipid/DNA complex	-	Influenza (H1N1)	whole inactivated IAV vaccine (H1N1, H3N2)	cationic lipid/DNA complex	Preclinical	IM	[177]

* Aquifex aeolicus lumazine synthase (LuS), ** respiratory syncytial virus fusion (F) *** SAS: Sigma Adjuvant System. Intra-Muscular (IM), Intra-Nasal (IN), Sub-Cutaneous (SC).

**Table 3:** Nano-vaccines developed for intranasal delivery in viral respiratory diseases.

Type of Nanoparticle	Main Material	Size (nm)	Target Respiratory Virus	Antigen/Epitope	Ref.
Polymeric	PLGA	225	Bovine parainfluenza 3 virus (BPI3V)	BPI3V proteins	[121]
	PLGA	200–300	Swine influenza virus (H1N2)	Inactivated virus H1N2 antigen	[122]
	γ-PGA	100–200	Influenza (H1N1)	Hemagglutinin	[123]
	Chitosan	140	Influenza (H1N1)	H1N1 antigen	[125]
	Chitosan	300–350	Influenza (H1N1)	HA-Split	[162]
	Chitosan	572	Swine influenza virus (H1N2)	Killed swine influenza antigen	[124]
	Chitosan	200–250	Influenza (H1N1)	M2e peptide	[164]
	HPMA/NIPAM	12–25	RSV	F protein	[165]
	PEG	40–500	RSV	F protein	[202]
	SA-CPH copolymer	348–397	RSV	Eα peptide	[208]
	CPH-CPTEG copolymer	-	RSV	F and G glycoproteins	[126]
Self-assembled proteins and peptides (SANP)	Nucleocapsid (N) protein of RSV	15	RSV	RSV phosphoprotein	[167]
	Nucleocapsid (N) protein of RSV	15	RSV	FsII epitope	[167]
	Nucleocapsid (N) protein of RSV	15	Influenza (H1N1)	M2e peptide	[168]
	Ferritin	12.5	Influenza (H1N1)	M2e peptide	[169]
	Influenza acid polymerase and the Q11 self-assembly domain	-	Influenza (H1N1)	Acid polymerase	[176]
Inorganic	gold	12	Influenza (H1N1, H3N2, H5N1)	M2e peptide	[88]
VLP	-	-	Influenza (H1N1)	Hemagglutinin	[143]
	-	80–120	Influenza (H1N1, H3N2, H5N1)	M2e5x peptide	[159]
	-	60–80	RSV	F protein et G glycoprotein of RSV and M1 protein of Influenza	[171]
Liposome	DLPC	30–100	Influenza (H1N1)	M2, HA, NP	[175]
Liposome, Polymer	10:1:1:1 of DPPC, DPPG, Cholesterol (Chol), and DPPE-PEG2000	89	SARS-COV 2	S+ STING agonist	[209]
LNP	ChAdenovirus (S)	-	SARS-COV 2	ChAd-S	[207]

1,6-bis(p-carboxyphenoxy) hexane (CPH); 1,6-bis-(p-carboxyphenoxy) hexane (CPH) anhydride; 1,8-bis(p-carboxyphenoxy)-3,6-dioxaoctane (CPTEG); Dilauroylphosphatidylcholine (DLPC); Matrix Protein 2 (M2e); Poly (D, L-lactide-co-glycolide, (PLGA); Poly-γ-Glutamic Acid (γ-PGA); Respiratory Syncytial Virus (RSV); Sebacic Anhydride (SA); Virus-Like Particle (VLP).

**Table 4:** Clinical phase SARS-CoV-2 vaccines.

Candidate Vaccine	Characteristics	Nano-Composition	Developer-Country	Status
mRNA-1273	mRNA vaccine encoding S protein	SM-102, PEG2000, Tromethamine,	Moderna/USA	FDA- EMA Approved
BNT162b2	mRNA vaccine encoding S protein	ALC-0315, ALC-0159, 1,2-distearoyl-sn-glycero-3-phosphocholine	Pfizer-BioNtech/USA-Germany	FDA-EMA Approved
Ad5-nCoV	Adenovirus type 5 vector that expresses S protein	LNP	CanSino Biologicals/China	China-Approved
AZD1222 (Covishield)	ChAdOx1-S		AstraZenca/UK-Sweden	FDA-EMA Approved
Ad26.COV2. S	Adenovirus type 26 vector that expresses S protein		Johnson & Johnson (Janssen)	FDA-Approved
INO-4800	DNA plasmid encoding S protein delivered by electroporation		Inovio Pharmaceuticals	Phase I (NCT04336410)
LV-SMENP-DC	DCs modified with a lentiviral vector expressing synthetic minigene based on domains of selected viral proteins; administered with antigen specific CTLs		Shenzhen Geno-Immune Medical Institute	Phase I (NCT04276896)
Pathogen-specific aAPC	aAPCs modified with a lentiviral vector expressing synthetic minigene based on domains of selected viral proteins		Shenzhen Geno-Immune Medical Institute	Phase I (NCT04299724)

aAPC: artificial antigen-presenting cell; CTL: cytotoxic T lymphocyte; DC: dendritic cell; LNP: lipid nanoparticle; S protein: SARS-CoV-2 spike protein [222].

## Abbreviations

ADE	antibody-dependent enhancement
APC	antigen-presenting cell
CpG	cytosine-guanine rich oligonucleotide
DC	dendritic cell
EMA	European Medicines Agency
FDA	Food and Drug Administration
hACE2	human angiotensin converter enzyme-2
HPMA/NIPAM	N-(2-hydroxypropyl) methacrylamide/N-isopropylacrylamide
IEDB	Immune Epitope Database and Analysis Resource
IFN-γ	interferon-g
IL	interleukin
MERS-CoV	Middle East respiratory syndrome coronavirus
NALT	nasal-associated lymphoid tissue
NLR	nucleotide-binding domain and leucine-rich repeat-containing
NLRP3	NLR Family Pyrin Domain Containing 3
PapMV	papaya mosaic virus
PEG	polyethylene glycol
PGA	poly-glutamic acid
pIgR	polymeric Ig receptor
PLGA	poly lactic-co-glycolic acid
RBD	receptor-binding domain
ROS	reactive oxygen species
RSV	respiratory syncytial virus
SAPNs	self-assembling protein and peptide nanoparticles
SARS	severe acute respiratory syndrome
VLPs	virus-like particles
CMV	Cytomegalovirus
